# HERV-W polymorphism in chromosome X is associated with multiple sclerosis risk and with differential expression of MSRV

**DOI:** 10.1186/1742-4690-11-2

**Published:** 2014-01-09

**Authors:** Marta García-Montojo, Belén de la Hera, Jezabel Varadé, Ana de la Encarnación, Iris Camacho, María Domínguez-Mozo, Ana Arias-Leal, Ángel García-Martínez, Ignacio Casanova, Guillermo Izquierdo, Miguel Lucas, Maria Fedetz, Antonio Alcina, Rafael Arroyo, Fuencisla Matesanz, Elena Urcelay, Roberto Alvarez-Lafuente

**Affiliations:** 1 Multiple Sclerosis Unit, Hospital Clínico San Carlos, Instituto de Investigación Sanitaria del Hospital Clínico San Carlos, Profesor Martin Lagos s/n., 28240, Madrid, Spain; 2 Immunology Department, Hospital Clínico San Carlos, Instituto de Investigación Sanitaria del Hospital Clínico San Carlos, Profesor Martin Lagos s/n., 28240, Madrid, Spain; 3 Multiple Sclerosis Unit, Hospital Virgen Macarena, Av. Dr. Fedriani, 3., 41071, Sevilla, Spain; 4 Molecular Biology Department, Hospital Virgen Macarena, Av. Dr. Fedriani, 3., 41071, Sevilla, Spain; 5 Instituto de Parasitologia y Biomedicina ’Lopez-Neyra’-CSIC, Parque Tecnológico de Ciencias de la Salud, Av. del Conocimiento s/n., 18016, Armilla (Granada), Spain

**Keywords:** Multiple sclerosis, Human endogenous retrovirus, HERV-W, Multiple sclerosis associated retrovirus, Chromosome x, Sex, Gender differences, Autoimmunity

## Abstract

**Background:**

Multiple Sclerosis (MS) is an autoimmune demyelinating disease that occurs more frequently in women than in men. Multiple Sclerosis Associated Retrovirus (MSRV) is a member of HERV-W, a multicopy human endogenous retroviral family repeatedly implicated in MS pathogenesis. MSRV envelope protein is elevated in the serum of MS patients and induces inflammation and demyelination but, in spite of this pathogenic potential, its exact genomic origin and mechanism of generation are unknown. A possible link between the HERV-W copy on chromosome Xq22.3, that contains an almost complete *open reading frame*, and the gender differential prevalence in MS has been suggested.

**Results:**

MSRV transcription levels were higher in MS patients than in controls (U-Mann–Whitney; p = 0.004). Also, they were associated with the clinical forms (Spearman; p = 0.0003) and with the Multiple Sclerosis Severity Score (MSSS) (Spearman; p = 0.016). By mapping a 3 kb region in Xq22.3, including the HERV-W locus, we identified three polymorphisms: rs6622139 (T/C), rs6622140 (G/A) and rs1290413 (G/A). After genotyping 3127 individuals (1669 patients and 1458 controls) from two different Spanish cohorts, we found that in women rs6622139 T/C was associated with MS susceptibility: [χ^2^; p = 0.004; OR (95% CI) = 0.50 (0.31-0.81)] and severity, since CC women presented lower MSSS scores than CT (U-Mann–Whitney; p = 0.039) or TT patients (U-Mann–Whitney; p = 0.031). Concordantly with the susceptibility conferred in women, rs6622139*T was associated with higher MSRV expression (U-Mann–Whitney; p = 0.003).

**Conclusions:**

Our present work supports the hypothesis of a direct involvement of HERV-W/MSRV in MS pathogenesis, identifying a genetic marker on chromosome X that could be one of the causes underlying the gender differences in MS.

## Background

Human endogenous retroviruses (HERVs) are believed to be remnants of ancient exogenous infections. They entered the germ line during primate evolution and as retroviruses were able to replicate and retrotranspose, increasing their copy number and spreading all over the genome. Indeed, approximately an 8% of the genome has a retroviral origin [1]. During evolution, most of the HERV proviruses have undergone extensive mutations and are unable to replicate. Nowadays, they reside in the genome of all human cells and are transmitted in Mendelian fashion.

Retroviruses typically consist of an internal region containing *gag, pro, pol,* and *env* genes, flanked by two long terminal repeats (LTR). Some HERVs have retained open reading frames (ORFs) putatively encoding functional proteins. These proteins can display a physiological role, as Syncytin-1 which is involved in the formation of the syncytiotrophoblast during pregnancy [2]; but as viral products, they might show antigenic properties involving the immune system. Indeed, HERVs have been associated with several pathologies as cancer and autoimmune diseases. HERVs RNAs, proteins or virions have been found in different tumor tissues [3-7], in schizophrenia [8-10], rheumatoid arthritis [11] and especially in multiple sclerosis (MS) [12,13].

Mainly two HERV families have been related to MS: HERV-W and HERV-H; and among them, the member most intensively studied is MSRV, a HERV-W-related retroelement [14].

Depending on the cohort studied, the percentage of MS serum or plasma samples positive to MSRV ranges from 50% [15] to 100% [16]. Enhanced expression of MSRV was also detected in MS plaques in independent post-mortem brain studies [17-19] and increased MSRV copy number in MS blood DNA was associated with a poorer MS prognosis [20,21]. Besides, interferon-beta reduces MSRV load in MS patients with a good clinical response to the treatment [22], suggesting that it could be a biomarker to monitor disease progression and therapy outcome.

MSRV might be exerting its role in MS pathogenesis through the activation of innate immunity and subsequent release of pro-inflammatory cytokines [23] through Toll-like receptor (TLR-4) [24]. It acts as a superantigen producing polyclonal T-lymphocyte activation [25] and it triggers a T-cell mediated neuropathology *in vivo*[26].

In spite of its pathogenic potential, the exact genomic origin of MRSV is unknown. When MSRV was discovered, controversy arose regarding whether it was an endogenous or exogenous virus [14]; at present, it seems that MSRV is most likely transcribed from an HERV-W endogenous element [27]. The HERV-W family consists of approximately 650 elements dispersed through the human genome [28], but the only member showing a full-length proviral copy with an ORF is the locus *ERVWE1* in the 7q21.2 chromosome, which encodes Syncytin-1 (Accession number: AF156963). *In silico* analyses have revealed that, in addition to the one previously described in chromosome 7, other putative complete retroviral units of HERV-W with *gag*, *pro*, *pol* and *env* might be replication competent [29]. Among all the *HERV-W* locations, the copy in chromosome Xq22.3 (*ERVWE2*) could be involved in MSRV expression due to several reasons. First of all, the *env* gene at this locus is the only known, besides *ERVWE1,* with an ORF only interrupted by a stop codon, and it has retained its coding capacity producing an almost complete N-truncated protein [30]*in vitro*. This sequence from chromosome X has important homologies with several reported MSRV clones derived from retrotranscription of RNA obtained from retroviral-like particles in MS patients. For example, the clone pV14 (AF331500) of MSRV *env* aligned with 98.5% of sequence identity and interestingly, the complete clone CL15 (AF127228) was 99.53% homologous to *env + pol* probably reflecting transcription of this region [27]. Moreover, a genetic origin in chromosome X could explain in part the higher prevalence of MS in women than in men (2:1 ratio).

In order to find polymorphisms associated with MS susceptibility that could be related with differential expression of MSRV in patients and controls, we mapped the *HERV-W* insertion in chromosome Xq22.3 and genotyped a total of 1669 patients and 1458 controls, from two different Spanish cohorts.

## Results

### Association of MSRV transcription levels with MS diagnosis and clinical evolution

**Figure 1:**
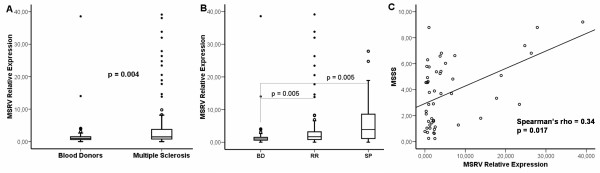
**Association of MSRV transcription levels with MS diagnosis and clinical evolution. A)** MSRV relative expression* was higher in MS patients (n = 112) compared to controls (n = 68) (U-Mann–Whitney; p = 0.004). **B)** MSRV relative expression* correlated with clinical forms (Spearman’s rho; p = 3*10^-4^). MSRV relative expression* in BD (n = 68) *vs.* RR patients (n = 81) (U-Mann–Whitney; p = 0.005), RR vs. SP patients (n = 15) (U-Mann–Whitney; p = 0.108) and BD *vs* SP patients (U-Mann–Whitney; p = 0.005). **C)** Correlation of MSRV relative expression* and MSSS score in women (n = 60) (Spearman’s rho = 0.34; p = 0.017). Subjects were randomly selected. *MSRV Relative Expression = 2e-∆∆Ct = 2e-((Ct MSRV – Ct GUSB)_sample_-Mean (Ct MSRV – Ct GUSB)_Blood Donors_).

To confirm the previously published association between MSRV RNA levels and MS, MSRV RNA expression was studied by RT-PCR in a randomly selected group of MS patients (n = 112; 55.6% women) and controls (n = 68; 53.3% women). The analysis was made with a set of primers and probe able to discriminate between syncytin-1 and MSRV env sequences [31]. MS patients presented higher MSRV RNA levels compared to controls (U-Mann–Whitney; p = 0.004) (Figure 1A). Also, we found a correlation with the clinical forms (Spearman’s rho = 0.28; p = 0.0003) and lower levels of MSRV expression were found in blood donors (BD) than in relapsing-remitting (RR) MS patients (U-Mann–Whitney; p = 0.005) or secondary-progressive (SP) patients (U-Mann–Whitney; p = 0.005) (Figure 1B).

Regarding clinical parameters, a correlation between MSRV relative expression and Multiple Sclerosis Severity Score (MSSS) [32] (Spearman’s rho = 0.25; p = 0.016) was observed; also the number of relapses suffered in the last two years tended to correlate with MSRV expression (Spearman’s rho = 0.19; p = 0.06). When we stratified by sex, the correlation coefficient between the MSRV expression and MSSS increased in women (Spearman’s rho = 0.34; p = 0.017) (Figure 1C), while only a trend could be seen in men (Spearman’s rho = 0.22; p = 0.16).

### Mapping of HERV-W copy in chromosome Xq22.3

**Figure 2:**
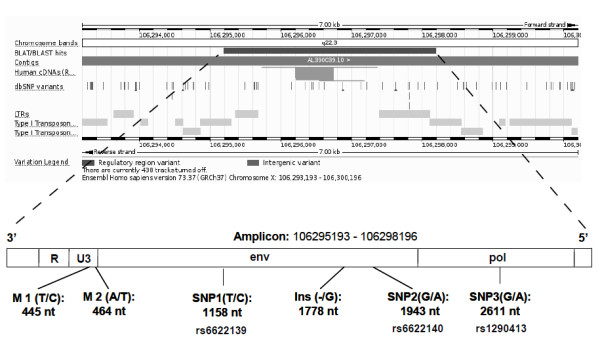
**Position of mapped amplicon in chromosome Xq22.3 (Ensembl Homo Sapiens version 66.37; March 2012) and schematic location of mutations (M1 and M2), insertion (Ins) and SNPs (rs6622139, rs6622140 and rs1290413), found in *HERV-W *** **region.**

First, we selected a region of 3 kb (106295193 – 106298196; Human GRCh37, March 2012) including the complete *HERV-W* locus. This sequence includes 3′ portions of the *pol* gene, the complete *env* gene, and the U3 and R regions of the 3′LTR (Figure 2). Then, we amplified the sequence through 18 overlapping amplicons. Each PCR product was analyzed by High Resolution Melting (HRM) curve analysis in 100 individuals, detecting 5 different mutations: T/C at 445 nt, A/T at 464 nt, T/C at 1158 nt, G/A at 1943 nt and G/A at 2611 nt from the beginning of the amplicon. Besides, an insertion of a G nucleotide was found at 1778 nt. Only 3 of these variants were detected in more than 5% of the individuals: T/C at 1158 nt position (SNP1: rs6622139), G/A at 1943 nt (SNP2: rs6622140) and G/A at 2611 nt (SNP3: rs1290413). The analysis in Haploview 4.0 showed that rs6622139 and rs6622140 were in perfect linkage disequilibrium (r^2^ = 1) and both map in the *env* open reading frame; therefore, only rs6622139 and the third polymorphism, rs1290413, were further studied in our cohort.

### Association of rs6622139 and rs1290413 with MS susceptibility

A total of 3127 individuals from two different Spanish cohorts were genotyped for rs6622139 and rs1290413: 893 MS patients and 664 controls from the discovery cohort (Madrid) and 776 MS patients and 794 controls from the replication cohort (Andalusia). Clinical and demographic characteristics of patients and controls are described in Table 1.

**Table 1:** Clinical and demographic characteristics of studied individuals

	***Discovery cohort***		***Replication cohort***	
***Characteristics***	***Patients***	***Controls***	***Patients***	***Controls***
Subjects (n)	893	664	776	794
Female (n (%))	593 (66.4)	371 (55.9)	539 (69.5)	513 (64.6)
Age (years) (Mean ± SD)	40.3 ± 9.5	41.2 ± 16.3	46.1 ± 11.4	40.2 ± 13.8
Age at onset (years) (Mean ± SD)	28.6 ± 8.1	-	30.8 ± 9.7	
Clinical form:				
RR (n (%))	595 (66.6)	-	610 (78.6)	-
SP (n (%))	51 (5.7)	-	152 (19.6)	-
PP (n (%))	64 (9)	-	60 (7.7)	-
Disease duration (years) (Mean ± SD)	11 ± 6	-	13.2 ± 9.2	-
Current EDSS score (Mean ± SD)	3 ± 2		n.a*	

*Not available.

The SNPs were analyzed separately in both sexes due to their location in chromosome X. Genotyping success rate was over 98% for both markers. Genotype distribution of the SNPs in controls and MS patients and p-values are shown in Table 2.

**Table 2:** Genotype frequencies and statistical significance for rs6622139 and rs1290413 in Chr.X- HERV-W env copy

**rs6622139 (T → C)**														
Females														
	Center of Spain						South of Spain							
	MS		Controls		p-value	OR (95% C.I)	MS		Controls		p-value	OR (95% C.I)	p _MH_	OR (95% C.I) _MH_
	n	%	n	%			n	%	n	%				
TT	413	70	245	66			353	65	338	66				
TC	167	28	108	29			170	32	148	29				
**CC**	**13**	**2**	**18**	**5**	0.02*	**0.44 (0.20 -0.96)**	**16**	**3**	**27**	**5**	**0.06**	**0.55(0.28-1.08)**	0.004**	**0.50(0.31-0.81)**
Males														
	Center of Spain						South of Spain							
	MS		Controls		p-value	OR (95% C.I)	MS		Controls		p-value	OR (95% C.I)	p _MH_	OR (95% C.I) _MH_
	n	%	n	%			n	%	n	%				
T-	251	84	253	86	0.36	1.23 (0.77-1.99)	194	82	213	76	0.09	0.69(0.44-1.09)	------	----------
C-	49	16	40	14			43	18	68	24				
**rs1290413 (G → A)**														
Females														
	Center of Spain						South of Spain							
	MS		Controls		p-value	OR (95% C.I)	MS		Controls		p-value	OR (95% C.I)	p _MH_	OR (95% C.I) _MH_
	n	%	n	%			n	%	n	%				
GG	331	56	229	62			296	55	270	53				
GA	220	37	127	34			196	36	203	39				
AA	42	7	15	4	0.05*	1.81(0.96-3.47)	47	9	40	8	0.59	1.13(0.71-1.79)	0.10	1.34(0.94-1.91)
Males														
	Center of Spain						South of Spain							
	MS		Controls		p-value	OR (95% C.I)	MS		Controls		p-value	OR (95% C.I)	p _MH_	OR (95% C.I) MH
	n	%	n	%			n	%	n	%				
G-	219	73	224	76	0.33	1.20(0.81-1.77)	173	73	208	74	0.79	1.05(0.70-1.59)	0.38	0.89(0.68-1.16)
A-	81	27	69	24			64	27	73	26				

*Statistically significant difference.

In the discovery cohort significant associations of rs6622139 and rs1290413 with MS were found in women. The homozygous mutant genotype of rs6622139 showed a protective effect, being more frequent in control than MS women [p = 0.02; OR (95% CI) =0.44 (0.20 – 0.96)] and the homozygous mutant genotype of rs1290413 also showed a nominal association with increased predisposition to MS in women [p = 0.05; OR (95% CI) = 1.81 (0.96-3.47)] (Table 2).

When data of a replication cohort were combined in order to increase the sample size and gain statistical power, the association of rs6622139*C in homozygosis was corroborated [p = 0.004; OR (95% CI) = 0.50 (0.31-0.81)] (Table 2).

### Association of rs6622139 with MS disability

**Figure 3:**
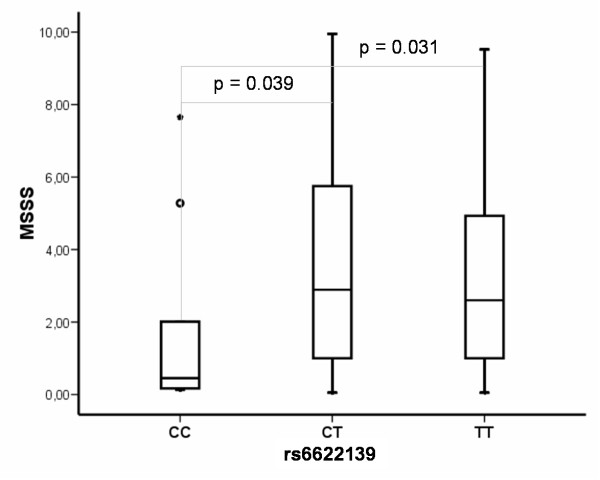
**Association of rs6622139 with MS disability in women.** Carriers of homozygous mutant genotype for rs6622139 (CC) presented lower MSSS scores than CT (U-Mann–Whitney; p = 0.039) or TT (U-Mann–Whitney; p = 0.031) carriers.

As rs6622139 was associated with MS susceptibility in women, we analyzed its role in clinical evolution. MSSS score was available from most patients from the Madrid cohort, thus the analysis was made including these subjects. Among MS women, carriers of the homozygous mutant genotype for rs6622139 (CC) (n = 9) presented lower MSSS scores [32] than either CT (n = 118) (U-Mann–Whitney; p = 0.039) or TT (n = 245) (U-Mann–Whitney; p = 0.031) carriers (Figure 3).

### Association of rs6622139 with MSRV transcription levels

**Figure 4:**
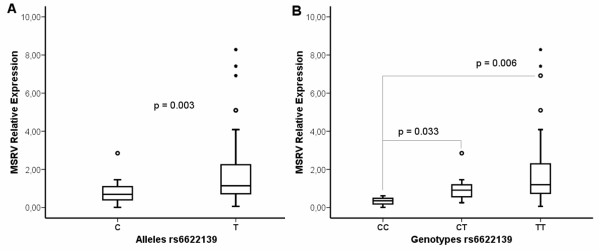
**Association of rs6622139 with MSRV transcription levels in women (MS patients and controls). A)** rs6622139*T allele (n = 136) was associated to a higher MSRV relative expression* than rs6622139*C allele (n = 22) (U-Mann–Whitney; p = 0.003). **B)** MSRV relative expression* according to rs6622139 genotypes (U-Mann–Whitney; CC (n = 3) *vs.* CT (n = 16): p = 0.03 and CC *vs*. TT (n = 60): p = 0.006). *MSRV Relative Expression = 2e-∆∆Ct = 2e-((Ct MSRV – Ct GUSB)_sample_-Mean (Ct MSRV – Ct GUSB)_Blood Donors_).

In agreement with the susceptibility conferred to women, rs6622139*T allele was associated with higher levels of MSRV RNA than rs6622139*C (U-Mann–Whitney; p = 0.003) in the women with available data analyzed (Figure 4A). Moreover, a dose-effect was evidenced from the association of rs6622139 genotypes and MSRV transcription levels (U-Mann–Whitney; CC *vs.* CT: p = 0.033 and CC *vs*. TT: p = 0.006) (Figure 4B). However, since the number of individuals with GG genotype in this group is very limited, any conclusion should be taken with caution.

## Discussion

In the present study we confirm increased levels of MSRV transcripts in MS patients compared to controls and correlation between MSRV expression and clinical severity (secondary progressive condition and MSSS) supporting the role of this element in MS pathology. Besides, we performed a genetic screening of the *HERV-W* locus in chromosome Xq22.3, identifying a polymorphism that is associated with MS susceptibility and severity in women. Finally, we found significant differences between levels of MSRV expression comparing carriers of the rs6622139 genotypes. All these findings support the role of chromosome Xq22.3 HERV-W locus in MSRV expression and MS etiopathogenesis.

The polymorphism rs6622139 associated with the disease is in perfect linkage disequilibrium (r2 = 1) with rs6622140 and both map in the orf of the *env* sequence. Taking MSRV env sequence (AF33150) as reference, the locus *ERVWE2* studied here presents a stop codon at position 39; however, this locus harbours an orf with a start codon (ATG) at position 68 that encodes for a N-terminally truncated 475 amino acid Env protein [30]. The N-truncated protein is expressed intracellularly *ex vivo*[30], while MSRV env, as a full-length retroviral envelope protein, is expected to be expressed at the cell surface. When the stop codon was reverted *in vitro* the resulting protein was expressed on the cell surface. Given the fundamental changes provoked by the elimination of the stop codon in ERVWE2, it has been speculated that MSRV env sequences could come from ERVWE2 variants lacking that stop codon [30]. After mapping ERVWE2 we did not find any mutation reversing the stop codon in our Spanish cohort.

Another hypothesis to explain the origin of the reported MSRV *env* sequences is that they might derive from recombination of transcripts from various HERV-W loci [27]. Apart from the full-length MSRV env, it has been also suggested that the N-truncated protein encoded by ERVW2 and other retroviral defective proteins may be expressed *in vivo* and exert functions in human physiology or pathology by themselves [30]. In fact, the primers used here and in other studies [31] that detect increased levels of HERV-W env (MSRV-type) RNA transcripts in MS patients compared to controls, would detect both the full-length MSRV *env* and the N-truncated *env* sequence encoded by ERVWE2, since they hybridize at the common C-terminal region. The SNPs rs6622139 (C/T) and rs6622140 (G/A) map at the residues 10 and 272, respectively, of the N-truncated protein (position 38 and 299 from the stop codon) and either the env protein is full-length or truncated, if ERVWE2 is involved in its origin, these polymorphisms would induce variations in its amino-acid sequence: rs6622139 (T/C) changes glycine by serine and rs6622140 (G/A) changes threonine by isoleucine. In both cases the substitution of an amino-acid with a hydroxyl (polar) group by an aliphatic one would be observed. This could affect the tridimensional configuration of the putative protein making epitopes more or less accessible to the immune cells, and therefore could alter its antigenic properties. On the other hand, changes in the nucleotide sequence of the RNA might affect its stability and thereby influence RNA levels.

The mechanism of generation of MSRV is unknown, but interestingly, complex interactions between herpesviruses, which have been historically studied for their involvement in MS pathogenesis, and HERVs have been brought to light. *In vitro*, Epstein-Barr virus (EBV) activates HERV-W/MSRV/syncytin-1 in cells derived from blood and brain [33] and antigens from other herpesviruses as HSV-1, HHV-6, and VZV are able to induce higher RT activity in peripheral lymphocytes from MS patients compared to controls [34].

It is well-known that MS is more prevalent in women than in men. Indeed, the female to male ratio for MS incidence currently ranges from 2:1 to 3:1 and varies by region [35]. Notably, over the past six decades, this ratio seems to have increased [36]. Beyond that, gender seems to influence other aspects of MS as age at onset [37], “parent-of-origin” effect on susceptibility [38] and risk for relatives of MS patients [38]. Although usually women do not have a poorer prognosis, higher levels of inflammatory lesions measured by MRI have been shown in female MS patients [39] and peripheral immune responses are more robust in females [40].

The relationship of MSRV with the gender differences in MS has already been suggested [20]. The DNA copy number of HERV-W (MSRV-type) is higher in MS patients than in controls, but it is specially increased in MS women. Indeed, a possible origin of MSRV in chromosome X is supported by the fact that the MSRV proviral load is higher in females than in men, not only among MS patients but also among controls [20]. Moreover, MSSS scores were higher among female patients with an elevated MSRV DNA load [20].

To our knowledge, this is the first demonstration of the genetic association with MS risk of the HERV-W *env* insert in chromosome X. The association supports the etiopathogenic role of this locus in MS risk, adding on previously published evidence.

The X chromosome has been proposed to constitute the common trait of the susceptibility to autoimmune diseases, other than to explain the female preponderance of these conditions [41]. A disease-promoting effect conferred by genes in the X chromosome has been suggested in females (since some genes escape X inactivation, resulting in double dosage in females) [41]. The critical role played by products of single genes located on the X chromosome or X-linked microRNAs in autoimmunity is evidenced also by the fact that most X-linked primary immune deficiencies carry significant autoimmune manifestations [41]. In this sense, the present data underline the crucial role of loci mapping to chromosome X in MS susceptibility.

## Conclusions

Our present work reinforces the hypothesis of a direct involvement of HERV-W/MSRV in MS pathogenesis, identifying a genetic marker on chromosome X that might contribute to the gender differences in MS. This sex-related difference could be exploited to develop novel therapeutic approaches for MS. Recently, the safety and pharmacokinetic profiles of GNbAC1, a humanized monoclonal antibody which is directed against MSRV-Env, have been evaluated and appeared favorable in healthy male volunteers [42]. In light of the present results, the next Phase II development program for this innovative therapeutic approach should include female patients as well, as an enhanced effect would be expected in them and would provide a genetic rationale for the gender bias observed in MS.

## Methods

### Patients and controls

A total of 1669 patients and 1458 controls from two different regions in Spain were included: from Madrid 893 MS patients (66.4% women) and 664 ethnically and age matched healthy controls (55.9% women) and from Andalusia 776 MS patients (69.5% women) and 794 controls (64.6% women); all of them had European ancestors. MS diagnosis was established according to McDonald’s criteria [43]. All individuals participating in this study signed informed consent and the study was approved by the Ethics Committees of the respective hospitals.

None of the control subjects reported any first or second degree relatives with any immunological disease. Clinical and demographic characteristics are enclosed in Table 1.

### Sequence data source

Previously published MSRV-env sequence (GenBank: AF33150) was used to map the HERV-W *env* element located on the negative strand of human chromosome Xq22.3. This sequence was aligned with the BLAST tool available at ensemble (http://www.ensembl.org) and enlarged 1500 nt at both 3′ and 5′ ends.

### Primers design

Using PRIMER3 Input version (0.4.0) software (http://frodo.wi.mit.edu/), a pair of primers specific for the sequences adjacent to MSRV in chromosome X as well as 18 pairs of primers to perform High Resolution Melting curve analysis (HRM) [44] in overlapping amplicons (Additional file 1: Table S1) were designed.

### PCR amplification of the HERV-W region at Xq22.3

The selected amplicon of 3004 bp (106295193 – 106298196; Human GRCh37, March 2012) including *HERV-W* locus in chromosome Xq22.3 was amplified by PCR using the above mentioned specific pair of primers (Additional file 1: Table S1). PCR reaction was performed on 40 ng of genomic DNA (35 cycles: 94ºC for 30s, 66ºC for 30 s and 68ºC for 180 s) in a final volume of 10ul of reaction mix containing the primers (0.4 uM), dNTPs (250uM), MgCl_2_ (2.5 mM) and 0.8 units of enzyme (BIO-X-ACT™ Long polymerase, Bioline). The amplification of the expected 3Kb fragment was confirmed by electrophoresis in 0.8% agarose gel.

### High Resolution Melting curve analysis (HRM)

The PCR product of the specific Chr. X region diluted 1/80 on sterile water was used as template to perform HRM curve analysis on a 7900 Fast Real-Time PCR System (Applied Biosystems), according to manufacturer’s recommendations. Over 1800 melting curves from 18 different amplicons were analyzed in 100 DNA samples (50 MS patients and 50 healthy controls).

### Sequencing analysis

At least three DNA samples from each group with altered melting curves compared with the average curve of wild-type subjects were sequenced. When a potential mutation or single nucleotide polymorphism (SNP) was detected, samples with the same melting curve were sequenced with BigDye Cycle sequencing kit (Applied Biosystems). Before sequencing, each HMR product was diluted 1/20 on sterile water.

### SNPs analysis

The newly described SNPs were analyzed by TaqMan assays by design, from Applied Biosystems in an ABI 7900HT Fast Real-Time PCR system (Applied Biosystems), following the manufacturer’s recommendations. The genotyping success rate was higher than 98%.

### MSRV expression analysis

Expression of MSRV was measured by quantitative real time RT-PCR with the relative method [45] which needs normalization with a housekeeping (HKG) gene. Before selecting Glucuronidase-B (*GUS-B)* as HKG gene, we analyzed the stability of its expression among MS patients and controls. The comparison of *GUS-B* Cts of both groups did not show significant differences (ANOVA, p = 0.98). Besides, efficiencies of both assays (*MSRV* and *GUS-B*) were tested by performing standard curves with serial dilutions of cDNA after retrotranscription of total human RNA treated with DNAse (Applied Biosystems); the efficiencies were 0.92 for *GUS-B* assay and 0.98 for *MSRV* assay.

RT positive controls and PCR controls were produced with the same batch of human total RNA (Applied Biosystems) as follows: part of the RNA diluted at 10 ng/ul was retrotranscribed into cDNA and then aliquoted to be used as “PCR control”; the rest of RNA was aliquoted to be used as “RT positive control” in each round of retrotranscription of our study. The cDNA was tested several times by real time PCR for MSRV and GUS-B and the means of the Cts were calculated. These values were used as references for the MSRV and GUS-B Cts of the “PCR controls” and the “RT positive controls” of each assay, since the same quantity of RNA was used in both cases. In case of failure, these two controls allowed us to detect at which step of the RT-PCR the problem occurred, i.e. if the retrotranscription step did not work correctly but the PCR did, the Ct of the PCR control would be similar to the reference value, but the Ct of the “RT positive control” would differ from them; on the contrary, if a problem occurred in the PCR step, both controls would differ from the reference value. PCR runs were accepted when both controls did not differ from the reference value more than 2% on Ct basis. This level of variation was used also to validate the duplicates of a sample [46].

From a group of randomly selected MS patients and blood donors RNA was extracted from PBMCs (Qiamp RNA Blood Mini kit. Qiagen) obtained by centrifugation of whole blood on CPT tubes (Becton Dickinson). The quality and concentration of RNA treated with DNAse (Applied Biosystems) were assessed by spectrophotometer. Then, sample volumes were adjusted to 10 ng/ul followed by two-step RT-PCR. Briefly, RNA was retrotranscribed with the First Strand cDNA Synthesis kit (Roche Diagnostics) with Oligo dT primers. In each round of retrotranscription, a positive control consisting of human total RNA with a known *MSRV* level of expression (RT positive control) and a “no RT” control of each sample, consisting of the same reaction mix without retrotranscriptase, were added. Then, a quantitative real time PCR was performed on the cDNA with a set of primers and a specific probe for *MSRV*[31] and for the HGK gene *GUS-B* (Applied Biosystems). Several controls were included in the PCR runs: 1) “No RT” control of each sample to detect possible DNA contamination; 2) RT positive control; 3) PCR control. Thus, the assays for the detection of *GUS-B* and *MSRV env* were considered acceptable in each sample when: 1) RT positive control and PCR control did not differ more than 2% on Ct basis; 2) Ct of HKG was lower than Mean + 2*S.D of HKG of all samples; 3) no amplification was detected in the “no RT” control; and 4) the duplicates of each sample had less than 2% of variability on Ct basis [46].

*MSRV* relative expression was measured by the 2e-ΔΔCt relative method [45]: MSRV Relative Expression = 2e-ΔΔCt = 2e-(ΔCt _sample_ – MeanΔCt_blood donors_) = 2e-((Ct MSRV – Ct GUSB)_sample_-Mean(Ct MSRV – Ct GUSB)_Blood Donors_). The mean of the Δ Ct of all the Blood Donors was used as the calibrator in all the expression analysis.

### Statistical analysis

Statistical analyses were performed using SPSS 15.0. Linkage disequilibrium values (D’) and Hardy-Weinberg equilibrium (H-W) were tested with Haploview 4.0 software. Chi-Square test (χ^2^) was used to compare allele and genotype frequencies. The Mantel–Haenszel (MH) test was used to perform the meta-analysis of allele and genotype frequencies of the two cohorts combined. MSRV relative expression was not normally distributed, therefore U-Mann Whitney test was used to compare MSRV relative expression between two groups. The non-parametric Spearman test was applied in order to evaluate the correlation between two variables. Statistically significant differences were considered when p-value < 0.05.

## Abbreviations

MS: Multiple sclerosis; MSRV: Multiple sclerosis associated retrovirus; HERVs: Human endogenous retroviruses; LTR: Long terminal repeat; ORF: Open reading frame; TLR: Toll-like receptor; BD: Blood donor; RR: Relapsing-remitting; SP: Secondary progressive; MSSS: Multiple sclerosis severity score; HRM: High resolution melting; CSF: Cerebrospinal fluid; EBV: Epstein-barr virus; HHV-6: Human herpesvirus-6; HKG: Housekeeping gene.

## Competing interests

The authors declare that they have no competing interests.

## Authors’ contributions

MGM participated in the design of the study, carried out the expression analysis, performed statistical analysis and drafted the manuscript. BH carried out the HRM analysis, genotyping, sequencing and performed statistical analysis. JV participated in the design of the study, designed the primers and performed statistical analysis. AE and IC performed part of the molecular genetics studies. MDM, AAL and AGM prepared the samples for genetic and expression studies. IC, GI, ML and RA collected clinical data and samples from the patients. MF, AA FM carried out genotyping of samples. RA, FM, EU and RAL have been involved in the conception and design of the study and supervised the work. All the authors have revised critically the manuscript and approved the final version.

## Supplementary Material

Additional file 1: Table S1. Primers used for mapping *HERV-W* region in chromosome Xq22.3. Click here for file
