# Supplementary material for: HERV-W polymorphism in chromosome X is associated with multiple sclerosis risk and with differential expression of MSRV
Source: Retrovirology. 2014 Jan 9;11:2. doi: 10.1186/1742-4690-11-2 (PMC3892049; doi:10.1186/1742-4690-11-2)
Supplement: Additional file 1: Table S1. — Primers used for mapping HERV-W region in chromosome Xq22.3. [file 1742-4690-11-2-S1.doc]

**Additional file 1: Table S1**: Primers used for mapping *HERV-W* region in chromosome Xq22.3.

| **Pair** | **Primer** | **Sequence** |
| --- | --- | --- |
| Xq22.3 pair | forward | TGCTGTATGTGTAAGGGCAGA |
|  | reverse | CATGCTGGCAGTATTCTGGA |
| Pair 1 | forward | GGGCTTTGTAGACTATGATAAGAAGTT |
|  | reverse | AGCACAGCAAGAGGGACAAT |
| Pair 2 | forward | GCTCGAATGCCTGGGTTTAT |
|  | reverse | CCCTAAGCCTAGCTGGGAAG |
| Pair 3 | forward | AGCCCCGTGTTTAAAGGTG |
|  | reverse | CCCTTGTTCCGATGTTAATGA |
| Pair 4 | forward | GGGTTGTGCAGTTGAGATTTCC |
|  | reverse | GCTTTACAGCTTCAATTCTGGAAG |
| Pair 5 | forward | GGCTCCATTTGAAGAACCAT |
|  | reverse | GGAGCTTCAAAACACCGAAC |
| Pair 6 | forward | AGAACCCAGGGCATCCAT |
|  | reverse | CCCTAGCAGCAGTAGTCCTTCA |
| Pair 7 | forward | AGAACCCAGGGCATCCAT |
|  | reverse | CCCTAGCAGCAGTAGTCCTTCA |
| Pair 8 | forward | TGTAGTAGAACTGAGTAGAGGTTGTGA |
|  | reverse | CAGCCTATCATTGTTTGAATGG |
| Pair 9 | forward | GGGGCACTAAGAATGAGAGG |
|  | reverse | GGACCTCTTGTTTCCAATCTG |
| Pair 10 | forward | CAGGTGAGGTTTGAGGTATGG |
|  | reverse | ACTCGGCTCCATGAGGTCT |
| Pair 11 | forward | GGAGGCACATCCAACAGTTA |
|  | reverse | GGAATTCAAGGTCAGGCAAG |
| Pair 12 | forward | CTATGTCCCCGGGTCAGTT |
|  | reverse | CCCGCAACTGCTATAACTCTG |
| Pair 13 | forward | CATTTTCCCTGTCCAATAATGA |
|  | reverse | CTCTTACCCCCTTTCGCTCT |
| Pair 14 | forward | GGTTGTACAGCAGCATGGAG |
|  | reverse | GGGAAGAGATCTTACTGTGTGGA |
| Pair 15 | forward | AGTGAGCATGCCGTTCACAT |
|  | reverse | TCCTTACACAGGTCCGAAGG |
| Pair 16 | forward | TCAATTTCCTTACTCAGGTACGC |
|  | reverse | GGAAACTCAGAAAGCCAATACC |
| Pair 17 | forward | CAGGTGCCCATCTTACTAAA |
|  | reverse | AAGAACGTATCCAGCCTACA |
| Pair 18 | forward | TGCTTTAGGGTTTTGGGATG |
|  | reverse | TTGCACAACATTGTGAATGTACTTA |
